# Composite Plate Rolling Technology of 304/Q345R Based on a Corrugated Interface

**DOI:** 10.3390/ma12233866

**Published:** 2019-11-23

**Authors:** Shun Wang, Guanghui Zhao, Yugui Li, Juan Li, Yaohui Song

**Affiliations:** Shanxi Provincial Key Laboratory of Metallurgical Device Design Theory and Technology, The Coordinative Innovation Center of Taiyuan Heavy Machinery Equipment, Taiyuan University of Science and Technology, Taiyuan 030024, Shanxi, Chinalyg060913@163.com (Y.L.); lijuanhello@163.com (J.L.);

**Keywords:** 304/Q345R, corrugated interface, bonding strength, numerical simulation

## Abstract

A new rolling process of the 304/Q345R composite plate based on a corrugated interface was developed. Through numerical simulation and rolling experiments, the metal deformation law, the stress and strain field distribution, and the bond strength of the corrugated plate were studied. By comparison, the corrugated interface effectively increased the length of the composite interface. Also, the composite interface from the 2D corrugated surface became a 3D corrugated surface, and a combination of the 304/Q345R composite plate metallurgy was achieved with a low reduction rate. Simultaneously, the interface bonding strength was improved.

## 1. Introduction

The composite plate in this work combines a carbon steel substrate upon which stainless steel is coated. Not only does it have the surface performance of stainless steel, but also, the mechanical strength and processing performance of carbon steel. Therefore, the composite plate can be widely utilized in chemical, marine, construction and food engineering, as well as in other fields. The composite plate that is produced through resource saving and cost reduction has attracted significant interest. It has been developed from a variety of methods. The mass production of the composite plate mainly includes the rolling composite method [1,2].

For the rolling production of a composite plate, researchers have carried out a large amount of research [3,4]. The reduction rate [5], rolling speed [6] and rolling temperature [7,8] effects on the composite plate have been the main aspects studied. Symmetry rolling [9], asynchronous rolling [10,11,12,13] and rolling through different temperatures have been used to solve the asymmetry problem caused by different plastic deformation of the two types of metals. The processing of surface oxide removal [14,15,16,17] and surface nano-crystallization for the composite interface were also key issues for the two-metal group of the billet interface.

Certain problems involving low bonding strength of the composite plate, low recombination rate, serious warp issues and high residual stress at the rolling stainless/carbon steel composite materials exist. This is because the two types of metals have significant differences in material mechanic performance. Therefore, the metal-interface bonding strengthening, the precision quality improvement, the rate of the composite, the good flatness and the efficiency improvement of industrial production are important problems.

Based on this engineering background, in this paper, a new corrugated rolling method of the 304/Q345R composite plate was proposed. The combination interface was a corrugated interface that increased the length of the bonded interface and changed the bonded interface from the traditional 2D flat interface to a 3D corrugated interface. The fabricated 304/Q345R composite with excellent corrugated interface demonstrated the interface strength improvement under a low reduction rate.

## 2. Material and Methods

### 2.1. Materials

**Figure 1:**
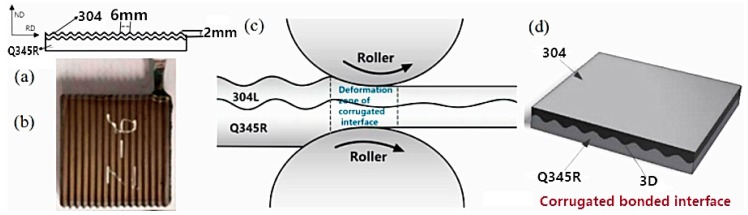
(**a**) The cross-sectional map of the rolled sample; (**b**) The preparation rolled plate; (**c**) Diagram of corrugated hot rolling; (**d**) Diagram of the rolled plate.

In this experiment, the Q345R steel was utilized as the base layer and the 304 stainless steel was utilized as the clad layer. The elements of the base layer and the clad layer are presented in Table 1. The mechanical properties of 304 stainless steel and Q345R steel are presented in Table 2. The corrugation in the experiment was processed through wire cutting. The 304 stainless steel is a parallel wave with an average thickness of 3 mm. The Q345R corresponding to the composite surface plate of stainless steel was corrugated. The lower surface of the Q345R container was a flat slab with an average thickness of 6 mm. As presented in Figure 1a, the wave height was 2 mm and the wave length was 6 mm.

The base plate and the composite compound surface were polished by a wire-drawing machine until the metal substrate was visible. The steel wire brush was utilized to polish the surface, for the sand surface effect to occur. Following, the polished surface was cleaned with anhydrous ethanol in order to remove the surface of the adhesive and oil. Subsequent to drying, argon arc welding was utilized to seal the composite slab and the vacuum nozzle was welded. Finally, a two-stage diffusion vacuum pump was utilized to vacuum the slab to the high vacuum state of 1.0 × 10^−3^ Pa. Figure 1b presents the rolled sample preparation. A diagram of corrugated hot rolling and a diagram of the rolled plate are shown in Figure 1b,c. Table 3 lists the rolling process parameters.

### 2.2. Experimental Observation

**Figure 2:**
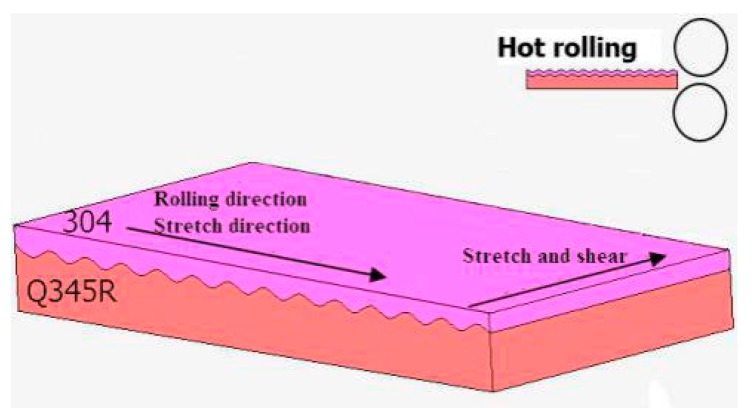
Sketch map of hot rolling.

The experiment was carried out with two-roll mill. The roll diameter was 306 mm. The rolling speed was 0.2 m/s. The temperature prior to rolling was 1150 °C. The rolling tests were executed under 25%, 30% and 40% reduction rates. The composite plate was processed by numerical-control wire cutting equipment. The tensile specimens were prepared. Following, the mechanical properties were tested by the WDW-200 universal tensile-testing machine (Jinan, Shandong, China). The mechanical tensile direction of the corrugated interface of the 304/Q345R rolling composite plate was parallel to the rolling direction. The direction of the shear specimen was perpendicular to the rolling direction. Figure 2 presents a schematic diagram of the mechanical testing.

### 2.3. Numerical Approach for Roll Bonding

In a complementary effort to understand the deformation and mechanism of the metal plastic processing, MSC Marc software was utilized to simulate the rolling process of the 304/Q345R composite plate with the corrugated interface. 2D plane strain conditions without lateral spreading were assumed. The plastic deformation behaviors of the 304/Q345R composite plate with the corrugated interface were compared with different reduction rates (r = 25%, 30% and 40%). The theoretical basis was provided for the rolling process development of the corrugated interface. The 304 stainless steel used X5CrNi18-9 in the material library of MSC MARC software (MSC Marc 2016, Los Angeles, CA, USA), corresponding to Chinese grade 0Cr19Ni9 stainless steel. The thermal expansion coefficient of the Q345R container steel was based on previous studies [18,19]. Other thermophysical properties of the Q345R container steel (including density, heat transfer, specific heat capacity, elastic modulus and Poisson’s ratio) were calculated using JMatPro software (JMatPro V9.0, UK).

The contact type of Q345R container steel (lower plate) was lower roll touching, and the friction coefficient was 0.2. The contact type of the 304 stainless steel (upper plate) and the upper-roll was touching, and the friction type was Coulomb friction. The friction coefficient of stainless steel during hot rolling was 30% to 50% higher than that of carbon steel, and the friction coefficient was 0.28. The heat transfer coefficient of the composite slab surface was defined as 10 W/(m^2^K), and the radiation factor was defined as 0.8 [20]. The conversion coefficient factor of the plastic deformation work during the rolling process to generate heat and frictional heat generation was defined as 0.9 [20]. The rolling rates were, respectively, 0.2 m/s. The ambient temperature was set to 20 °C during the composite rolling process, and the roll temperature was also 20 °C.

When the two-layer metal composite plate is rolled by a single metal plate, certain commonly used methods are added based on the following:

- To set the roller as a rigid roll and ignore the corresponding elastic deformation;
- To set the 304 and Q345R as an isotropic material;
- Setting the environmental temperature during the composite rolling at 20 °C;
- The 304/Q345R hot-rolled composite slab is welded.

In the interface between the substrate and the composite plate, it is not easy to produce defects and it is quite difficult to produce interface slip during rolling. Also, during hot-rolling plastic deformation, the substrate and the clad plate were in a viscous state. Therefore, the bonding between the substrate and the clad plate in the simulation was glue.

e. In order to roll the composite slab into roll gap, a push plate was added at the tail end of the composite slab. The push plate speed was lower than the rolling speed.

The sine curve was utilized as the interface corrugation. The wave hollow was 2 mm and the wavelength was 6 mm. The main parameters of the finite element were set as presented in Table 4.

## 3. Results and Discussion

**Figure 3:**
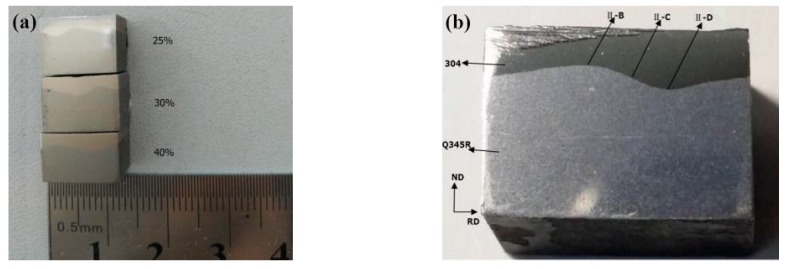
(**a**) RD-ND section of rolled composite plate under different reduction ratios; (**b**)The corrugated interface.

The finished composite plate was cut into small samples through wire cutting, and the RD-ND section was polished to observe the compound effect of the composite interface. Figure 3a presents the RD-ND section of the composite plate under different reduction rates, where the upper part was the 304 stainless steel plate and the lower part was a Q345R container plate. The corrugated composite interface was formed between the former plates. Figure 3b presents the magnification of the corrugated interface. The II-B, II-C and II-D symbols represent the peak, waist and valley, respectively. It can be observed that the composite plate formed a 3D composite corrugated interface. The MUT600 ultrasonic flaw detector was utilized to detect the composite plate. No composite area was observed and the composite rate of the corrugated interface was 100%.

### 3.1. Analysis of Tensile Mechanical Property

**Figure 4:**
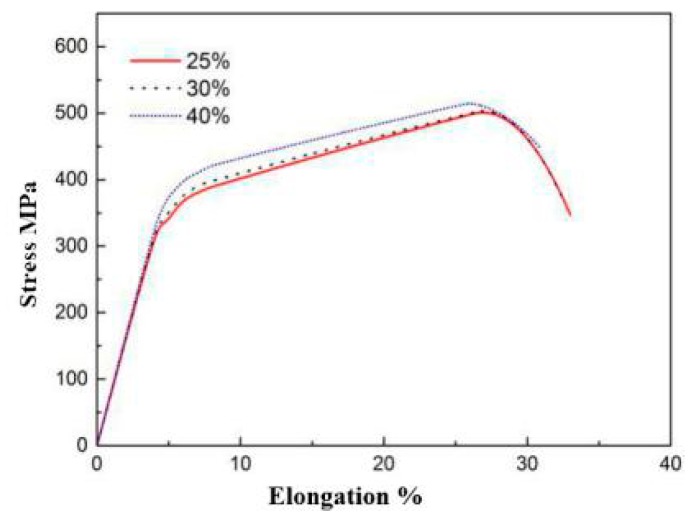
304/Q345R tensile curve of composite interface with different reduction ratios.

According to the relevant provisions of the Chinese standard GB/T228, the composite plate was produced through wire cutting. The tensile displacement rate was 1 mm/min. Subsequent to the sample cutting, the Zeiss SEM scanning electron microscope (SIGMA, Germany) was used to observe and analyze the fracture morphology and the fracture behavior. Figure 4 presents the tensile curves of the composite plate under different compression ratios. It can be observed that in the three different reduction rates, the tensile process was not observed in the yield oscillation stage, whereas it directly entered the intensive stage. The tensile strength exceeded 500 MPa in the 40% rolling reduction rate, displaying the two metals with a combination of higher status.

**Figure 5:**
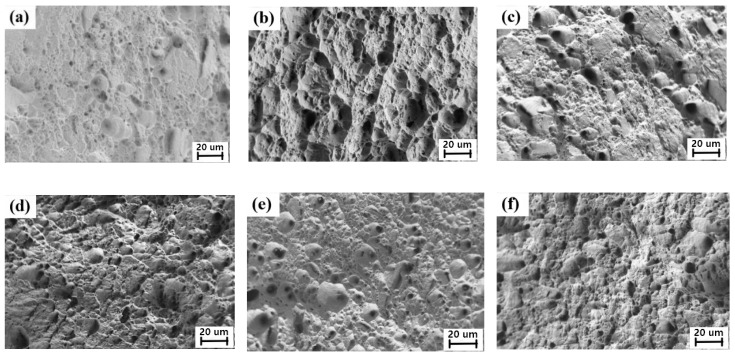
SEM morphology of tensile fracture at 304/Q345R corrugated interface; (**a**) 25% reduction ratio in 304 side; (**b**) 25% reduction rate in Q345R side; (**c**) 30% reduction ratio in 304 side; (**d**) 30% reduction rate in Q345R side; (**e**) 40% reduction ratio in 304 side; (**f**) 40% reduction rate in Q345R side.

Figure 5 presents the SEM fracture surface of the 304/Q345R composite plate with the corrugated steel interface under three reduction rates. The analysis was executed on the sides of the 304 stainless steel and the Q345R steel. As it can be observed from Figure 5, a high number of small-sized and dense dimples existed on the fracture surface of the side of the 304 steel and the Q345R. This was a significant feature of ductile fracture. The phenomenon indicated that the 304 and the Q345R steels sustained apparent plastic deformation prior to fracture. As the rolling reduction rate increased, the dimples became smaller in size.

### 3.2. Shear Performance Analysis

**Figure 6:**
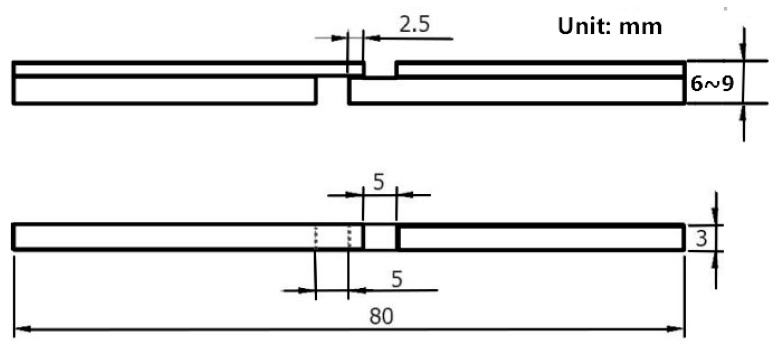
Dimension drawing of shear specimen.

In this paper, the bonding strength of the composite interface was measured through shear testing. In view of the characteristics of the corrugated interface of the corrugated composite plate, the peak, the waist and the valley sustained tensile–shear testing. Figure 6 presents a schematic diagram of the tensile–shear specimen.

**Figure 7:**
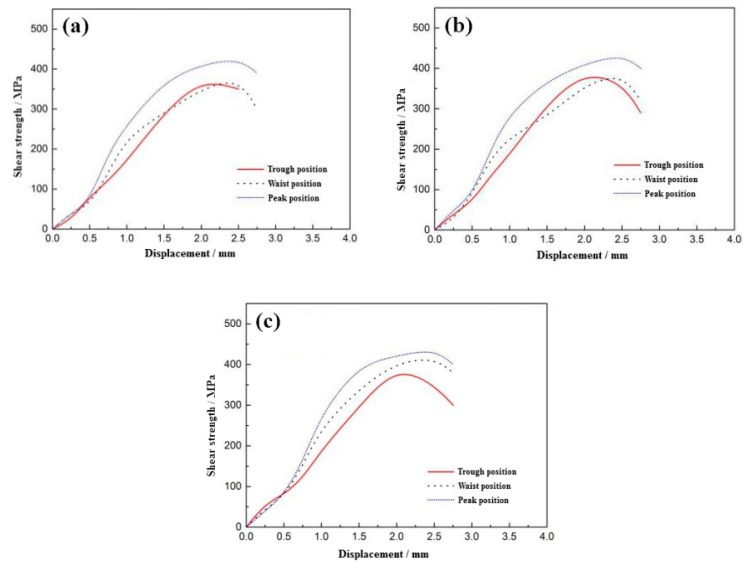
Shear curves of the 304/Q345R composite interface: (**a**) 25%, (**b**) 30%, (**c**) 40%.

Figure 7 presents the shear curve of the 304/Q345R composite interface under different reduction rates. As it can be observed from the graph, as the rolling reduction rate increased, the shear strength of the interface was improved. The shear strength of the peak position of the composite interface was the highest, whereas the valley position was the lowest. As the rolling reduction ratio increased, the difference in shear strengths between the peak position and the valley position was gradually reduced. When the reduction ratio was 40%, the difference was 41.03 Mpa. Under the same rolling reduction rate, the actual reduction rate was the highest in the peak position. Consequently, the shear strength was the highest.

Table 5 presents the shear strength of the 304/Q345R rolling composite interface under different reduction rates. It can be observed that the shear strength of the stainless steel clad plates at different positions was higher compared to the national standard of 210 MPa, meeting the provisions of the Chinese standard GB/T 8165-2008.

### 3.3. Simulation Results and Analysis

The finite-element simulation results of MSC Marc displayed that the equivalent strain and equivalent stress could demonstrate the deformation law of the metal and the flow law of the metal material, and determine the yield process as well as the order of the yield. When the ratio of equivalent stress of the deformation zone to the yield stress at current temperature exceeds 1, plastic deformation of the metal can occur. In this paper, the equivalent strain, the equivalent stress and the ratio were analyzed. The plastic deformation for different rolling interfaces was studied.

Numerical simulation was carried out for the three reduction rates of 25%, 30% and 40%, respectively. The results were as follows.

#### 3.3.1. Equivalent Plastic Strain Analysis

**Figure 8:**
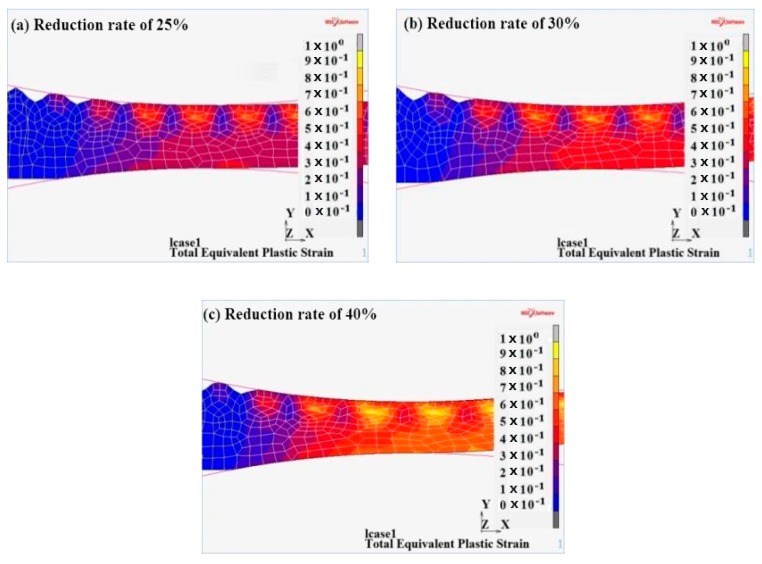
Equivalent plastic strain at different reduction rates.(**a**) 25%; (**b**) 30%; (**c**) 40%.

The wave peak touched the roll first and consequently, plastic deformation occurred, leading to the metal composite. As it can be observed from Figure 8, when the reduction ratio was 25%, the maximum equivalent effect was approximately 0.6 and the equivalent effect of the valley was approximately 0.2. When the reduction ratio was 30%, the maximum equivalent effect was approximately 0.65 and the equivalent effect of the valley was approximately 0.25. When the reduction ratio was 40%, the maximum equivalent effect was approximately 0.85 and the equivalent effect of the valley was approximately 0.45.

#### 3.3.2. Von Mises Equivalent Stress Analysis

**Figure 9:**
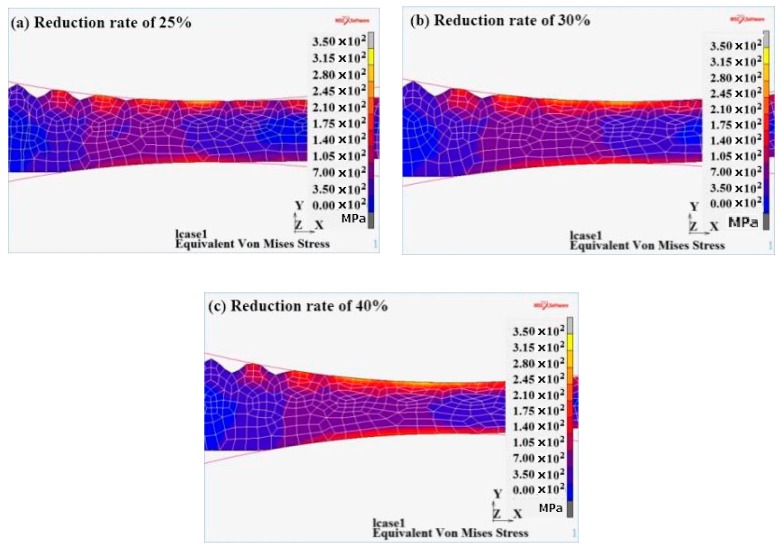
Von Mises equivalent stress Equivalent plastic strain at different reduction rates. (**a**) 25%; (**b**) 30%; (**c**) 40%.

As it could be observed from Figure 9, the equivalent-stress value of the contact surface was higher. The equivalent-stress value of the 304 exceeded the 345R equivalent-stress value, which was due to the high deformation resistance of the 304 stainless steel. When the deformation occurred, the equivalent-stress value was higher. The equivalent stress at the peak of the corrugated composite interface was the maximum. The equivalent stress at the valley was the minimum.

#### 3.3.3. Ratio of Equivalent Stress of Deformation Zone to Yield Stress at Current Temperature Analysis

**Figure 10:**
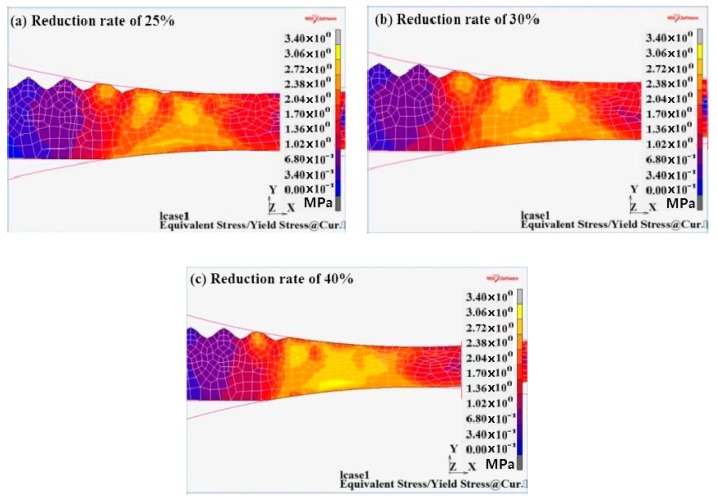
Ratio of equivalent stress of deformation zone to yield stress at current temperature at different reduction rates. (**a**) 25%; (**b**) 30%; (**c**) 40%.

As it can be observed from Figure 10, the ratio appeared at the peak of the corrugated interface and the region near the lower roller in the substrate Q345R steel under the three reduction rates. This was caused by the fact that the actual reduction rate of the wave peak of the corrugated interface was high and the substrate Q345R was easy to yield. The ratio exceeding 2.5 of the region continued to the heart of the composite plate. With the increase in the rolling reduction rate, the ratio of equivalent stress for the composite interface increased and the ratio area increased. When the corrugated interface was rolled, the range of the plastic yielding of the metal became increasingly higher, which was beneficial to the high deformation of the two metals in the roll gap. High plastic deformation is easy to develop into the composite interface, which could effectively promote the metallurgical bonding of the two metals.

## 4. Conclusions

A new rolling process of the 304/Q345R composite plate based on a corrugated interface was developed. The conclusions were as follows:

(1) The corrugated interface could effectively increase the length of the composite interface, in order for the composite interface from the 2D corrugated surface to become a 3D corrugated surface. No composite area was observed and the composite rate of the corrugated interface was 100%.

(2) Moreover, the tensile strength exceeded 500 MPa in the 40% rolling reduction rate. The 304 and the Q345R steels sustained apparent plastic deformation prior to fracture. As the rolling reduction rate increased, the dimples became smaller-sized.

(3) Through shear testing, it could be seen that the shear strength at different positions of the corrugated interface under three reduction rates was higher than the Chinese standard. As the rolling reduction ratio increased, the difference in shear strengths between the peak and trough positions gradually decreased.

(4) The numerical simulation results demonstrated that the plastic yielding of the metal increased due to the corrugated-interface rolling. This range contributed to the two-metals coordination of the high deformation in the roll gap. High plastic deformation was easy to develop into the composite interface, which could effectively promote the metallurgical bonding of the two metals.

## Figures and Tables

**Table 1:** Chemical composition of composite and base materials (mass fraction%).

Element	C	Si	Mn	P	S	Cr	Ni
304	0.04	0.33	1.37	0.037	0.005	18.3	8.1
Q345R	0.13	0.44	1.52	0.013	0.004	—	—

**Table 2:** Mechanical properties of 304 stainless steel and Q345R steel.

	Yield Stress/MPa	Ultimate Tensile Strength/MPa	Elongation %
304	205	≥515	40
Q345R	345	510–640	21

**Table 3:** Process table of corrugated hot rolling.

Roller Diameter (mm)	Rolling Temperature (°C)	Rolled Direction	Reduction (%)			Rolling Speed (m/s)
306	1150	Vertical texture	25	30	40	0.2

**Table 4:** Main parameters of the finite element method.

Name	Value
Coefficient of friction (Coulomb friction type)	0.28 between roll and stainless steel; 0.2 between roll and carbon steel
Radiation factor	0.8
Surface heat transfer coefficient/W/(m^2^K)	10
Rolling rates	0.2 m/s
Roll temperature	20 °C

**Table 5:** Shear strengths of the 304/Q345R composite interface under different reduction rates (unit MPa).

Reduction Ratio	Trough Position	Waist Position	Peak Position
25%	376.46	380.97	427.24
30%	392.72	389.08	435.87
40%	398.07	417.66	439.10
